# Average and individual differences between the 12-item MOS Short-form Health Survey version 2 (SF-12 V.2) and the veterans RAND 12-item Health Survey (VR-12) in the Chinese population

**DOI:** 10.1186/s12955-022-02010-z

**Published:** 2022-07-02

**Authors:** Daniel Y. T. Fong, Bobo K. Y. Chan, Sha Li, C. H. Wan, Lewis E. Kazis

**Affiliations:** 1 School of Nursing, Li Ka Shing Faculty of Medicine, The University of Hong Kong, 3 Sassoon Road, Hong Kong, China; 2 School of Humanities and Management, Research Center for Quality of Life and Applied Psychology, Key Laboratory for Quality of Life and Psychological Assessment and Intervention, Guangdong Medical University, Dongguan, China; 3 Department of Physical Medicine & Rehabilitation, Harvard Medical School, Spaulding Rehabilitation Hospital, Charlestown, MA USA; 4 School of Nursing, Nanjing Medical University, Nanjing, China; 5 Department of Health Law, Policy and Management, Boston University School of Public Health, Boston, USA

**Keywords:** Health-related quality of life, SF-12v2, VR-12, Individual differences, Chinese

## Abstract

**Background:**

The 12-item MOS Short-form Health Survey version 2 (SF-12v2) and the Veterans RAND 12-item Health Survey (VR-12) are generic health-related quality of life measures. They are fairly similar, but their differences in scores have not been assessed. Therefore, this study aimed to assess the differences between the SF-12v2 and the VR-12 in a Chinese population.

**Methods:**

We conducted a household survey of 500 Chinese adults in Hong Kong. Both the SF-12v2 and the VR-12 were self-administered. The physical component summary score (PCS) and the mental component summary score (MCS) of each instrument were computed using well established algorithms. Their mean differences were assessed using 95% confidence interval (CI), and their individual differences were assessed by Bland–Altman analysis.

**Results:**

The participants had a mean age of 38 years (range: 18–80 years). The mean PCS and MCS scores of the SF-12v2 were 50.3 (*SD* = 6.5) and 49.0 (*SD* = 9.0), while those of the VR-12 were 49.6 (*SD* = 6.2) and 49.7 (*SD* = 8.8), respectively. The corresponding paired differences (SF-12v2—VR-12) of the PCS and MCS were 0.8, 95% CI (0.4–1.1) and − 0.7, 95% CI (− 1.2 to − 0.2), respectively. All confidence limits fell within the minimal clinical important difference (MCID) of 3. The 95% limits of agreement were − 7.0, 8.5 for PCS and − 11.2, 9.9 for MCS, which fell outside the corresponding MCID for individual responses.

**Conclusion:**

The SF-12v2 and the VR-12 reached mean equivalence at the group sample level, but there was a range of individual differences.

## Background

Self-reported outcome instruments are used worldwide to measure health-related quality of life (HRQL). The 12-item Medical Outcomes Study (MOS) Short-form Health Survey (SF-12) and the 12-item Veterans RAND Health Survey (VR-12) are two generic instruments used to assess quality of life in the general population. The MOS SF-12 is a proprietary instrument, while the VR-12 is regarded as a low-cost alternative. Both instruments were derived from the RAND SF-36 Health Survey, which was developed in 1988 as part of the Medical Outcomes Study (MOS) [1, 2]. The RAND SF-36 Health Survey, also known as the MOS SF-36 Health Survey, comprises 36 items. Based on these items, two component scores are measured, namely the physical summary component score (PCS) and the mental component summary score (MCS); the items cover eight scales designed to assess various aspects of quality of life in the general population, namely physical functioning (PF), role limitations due to physical problems (RP), bodily pain (BP), general health perceptions (GH), vitality (VT), social functioning (SF), role limitations due to emotional problems (RE), and mental health (MH) [3]. Since then, the two instruments have undergone development separately. Thus, they are similar but have differently worded questions and different scoring algorithms.

The MOS SF-12 is a shortened version of the MOS SF-36 Health Survey that reproduces the PCS and MCS scores of the MOS SF-36 [3, 4]. The development of the SF-36v2 Health Survey in 1996 improved the clarity of the original item wording, changed dichotomous choices for seven items in the RP and RE scales to five choices, and removed a response option from items in the MH and VT scales [1]. These same changes were applied to the corresponding items in the MOS SF-12, resulting in the SF-12v2 Health Survey [5, 6]. On the other hand, the VR-12 was derived from the Veterans RAND 36-item Health Survey (VR-36), which was modified from the MOS SF-36 by increasing the response choices for RP and RE items to five-point Likert choices [7, 8]. The VR-12 also reproduces the PCS and MCS of the VR-36 and comprises the same eight scales of the MOS SF-36 [7, 9].

Both the MOS SF-36v2 and the VR-36 have demonstrated adequate measurement properties in the general population [1, 10], and the PCS and MCS reproduced from their short forms, SF-12v2 and VR-12, have also been commonly used in general population studies [8, 11]. The VR-12, in particular, has shown that it can be accurately linked to the global health scale of the Patient-Reported Outcome Measurement Information System (PROMIS) [12]. Although the SF-12v2 and the VR-12 basically comprise the same set of 12 items, there are differences in the item wording, response choices, and scoring (Table 1). Specifically, there are differences in the item wording. Moreover, the order of the response choices for the two RP items and the two RE items of the SF-12v2 are reversed in those of the VR-12 [13]. In addition, the two MH items and the VT item of the SF-12v2 have five response choices, whereas those of VR-12 have six response choices.

**Table 1:** Comparisons on the contents of the Chinese versions of the SF-12v2 and VR-12

Item	Scale	Contents	SF-12v2 versus VR-12	
			Item wording and response options	Scale scoring
1	General health	General health condition	Identical item wording^a^ and response options	The item was coded differently
2a	Physical functioning	Limitations on daily activities	•Different wording but same contents •Same number of response options •Slight differences in the wording of response labels	Same scoring as the average of the two items
2b		Moderate activities	The VR-12 included an example of playing golf, whereas the SF-12v2 used practicing Tai-Chi instead	
		Climbing stairs	Identical item wording^a^	
	Role physical	Problems due to physical health	•Different wording but same contents •Same number of response options •Slight differences in the wording of response labels but the order was opposite to each other	Reverse code both items of VR-12 but not for SF-12v2, and need different scoring
3a		Accomplished less than expected	Identical item wording^a^	
3b		Limitation in work or related activities	Identical item wording^a^	
	Role emotion	Problems due to emotions	•Different wording but same contents •Same number of response options •Slight differences in the wording of response labels but the order was opposite to each other	Reverse code both items of VR-12 but not for SF-12v2, and need different scoring
4a		Accomplished less than expected	Identical item wording^a^	
4b		Limitation in work or related activities	Identical item wording^a^	
5	Bodily pain	Influence on daily work due to pain	Same item wording, but the VR-12 specifically mentioned that daily work included occupational work and housework	Same scoring
		Feeling of specific conditions	•Different wording but same contents •5 responses options in SF-12v2, and 6 in VR-12	
6a	Mental health	Feeling calm	Identical item wording^a^	•Reverse code item 6a for both the SF-12v2 and VR-12 •Need different scoring
6c		Feeling bad mood	Identical item wording^a^	
6b	Vitality	Feeling energetic	Slight difference in item wording	•Reverse code the item for both the SF-12v2 and VR-12 •Need different scoring
7	Social functioning	Limitations in social activities due to physical or emotional problems	Slight differences in item wording and response labels	Same scoring

^a^“Identical item wording” refers to their identical item stems. The order and content of response categories across forms may also differ or be reversed

Apart from format differences, the SF-12v2 and the VR-12 also have different scoring procedures despite both procedures being standardized by the norms from a general population in the United States (US). First, they do not share the same item coding. Second, the SF-12v2 uses proprietary norms, collected in 1998 and 2009, whereas the VR-12 uses the non-proprietary 1990 US population norms, which have been updated in 2009 and 2018 [14, 15].

Although the PCS of the MOS SF-12 has shown a strong correlation with that of the VR-12 [16], item format and type have been shown to affect the psychometric properties, such as the internal reliability and structural validity, of an instrument [17, 18]. Moreover, the MOS SF-12 and the VR-12 have been shown to have distributional differences in the US population [19]. A similar result was observed in the German versions of the MOS SF-12 and the VR-12 [20]. Moreover, the PCS and MCS of the SF-12 were derived from orthogonal rotation, whereas those of the VR-12 were derived from oblique rotation [19]. In view of the differences, scores of the SF-12v2 and VR-12 in an US population have been linked [21, 22]. However, there were no direct comparisons made between the PCS and the MCS of the SF-12v2 and those of the VR-12 in a Chinese population. Estimating their differences would help to assess whether there are discernible differences between the two instruments. If not, the VR-12 would be a viable alternative to the SF-12v2. Therefore, this study aimed to directly compare the two instruments in a Chinese population.

## Methods

### Design and participants

This study was part of a cross-sectional household survey. The details of the survey have been described elsewhere [23–25].

Between February 2018 and September 2019, we recruited 500 participants who were 18 or older. We excluded those who had hearing problems, sleep problems sleep disturbances, or psychiatric illnesses. They were sampled from a representative sampling frame purchased from the Hong Kong Census and Statistics Department, which covered the entire territory. All participants signed an informed consent form before they completed the self-administered study questionnaires.

Ethics approval was obtained from the Institutional Review Board of the University of Hong Kong/Hospital Authority Hong Kong West Cluster (UW 17-011).

### Measurements

The sociodemographic questionnaire, the SF-12v2, and the VR-12 were administered in paper form. Specifically, each participant first completed the SF-12v2, followed by four survey modules that comprised 52 items, and then the VR-12. Other self-reported instruments that were administered have been described elsewhere [23–25].

The sociodemographic data collected were age, sex, marital status, education level, occupation, and chronic illnesses.

The standard Chinese version of the SF-12v2 was self-administered [26, 27]. The SF-12v2 had been well tested with satisfactory psychometric performance in both the adult and adolescent populations [26, 27]. It can be scored as eight scales and as the PCS and the MCS scores. They were scored in accordance with its scoring manual, using normative data from the 1998 US population for norm-based scoring [28]. The PCS and the MCS typically were normed to a US population with a higher score indicating a better quality of life in the corresponding scale/component.

The Chinese version of the VR-12 was also administered [29]. It was obtained from rigorous forward–backward translation, taking account of cultural differences, and had been psychometrically evaluated [29]. We obtained the R code from the developer for scoring using the 1990 US normative data [9]. The PCS and the MCS also were standardized and normed to a US population, with a higher score indicating a better quality of life in the corresponding component [30]. The eight scales were not normed but were standardized in the range of 0–100.

### Statistical analysis

The eight scales and the two components of the SF12-v2 and the VR-12, as well as the sample characteristics were summarized using descriptive statistics. We first assessed the average differences between the PCS, the MCS, and the eight scales of the SF12-v2 and the VR-12 by obtaining a 95% confidence interval (CI) for each component’s paired difference. The equivalence of a component was verified if the corresponding 95% CI was within its ± minimal clinically important difference (MCID). For the general population, the MCID was 3 for both the PCS, the MCS, and the eight scales [31, 32]. We also assessed the individual agreement between the two components by conducting a Bland–Altman analysis [33]. The 95% limits of agreement were obtained by the mean ± 1.96 standard deviation (SD) for the paired differences, which covered 95% of the differences [34]. Individual differences were verified if the agreement fell within the ± MCID for individual responses that accounted for the test precision. For the PCS, the MCID for individual responses was 6, whereas for the MCS it was 7 [31].

Linear regression was applied to examine the factors associated with the differences between the PCS and the MCS of the two instruments. Model adequacy was assessed by examining the model residuals. All statistical analyses were performed using R version 4.0.3 (R Foundation for Statistical Computing, Vienna, Austria).

## Results

Table 2 summarizes the sociodemographic characteristics of the participants (*N* = 500). The average age of the participants was 39 years (*SD* = 12; range = 18–80), 332 (66%) participants were female, 307 (61%) were married/cohabiting, 250 (82%) had a secondary school education, 370 (74%) were in the workforce, and 410 (82%) did not have any long-term illnesses.

**Table 2:** Sociodemographic information of 500 participants

Social-demographic variables	Mean ± SD/n	%
Mean age ± standard deviation	39 ± 12	
Sex		
Male	168	33.6
Female	332	66.4
Marital status		
Single	171	34.2
Married/Cohabiting	307	61.4
Separated/Divorced/Widowed	22	2.6
Educational level (1 missing data)		
Primary school or below	24	4.8
Secondary	251	50.3
Bachelor or above	224	44.8
Occupation		
Employed/In the working force	370	74.0
Not in working force	130	26.0
Chronic Illness		
Yes	90	8.0
Allergic Bowel Syndrome	1	0.2
Anxiety	10	2.0
Depression	10	2.0
Diabetes	9	1.8
Eczema	28	5.6
Gastric Ulcer	6	1.2
Hearing Problems	4	0.8
Heart Disease	6	1.2
High Cholesterol	24	4.8
Hypertension	22	4.4
Insomnia	3	0.6
No	410	92.0

**Fig. 1:**
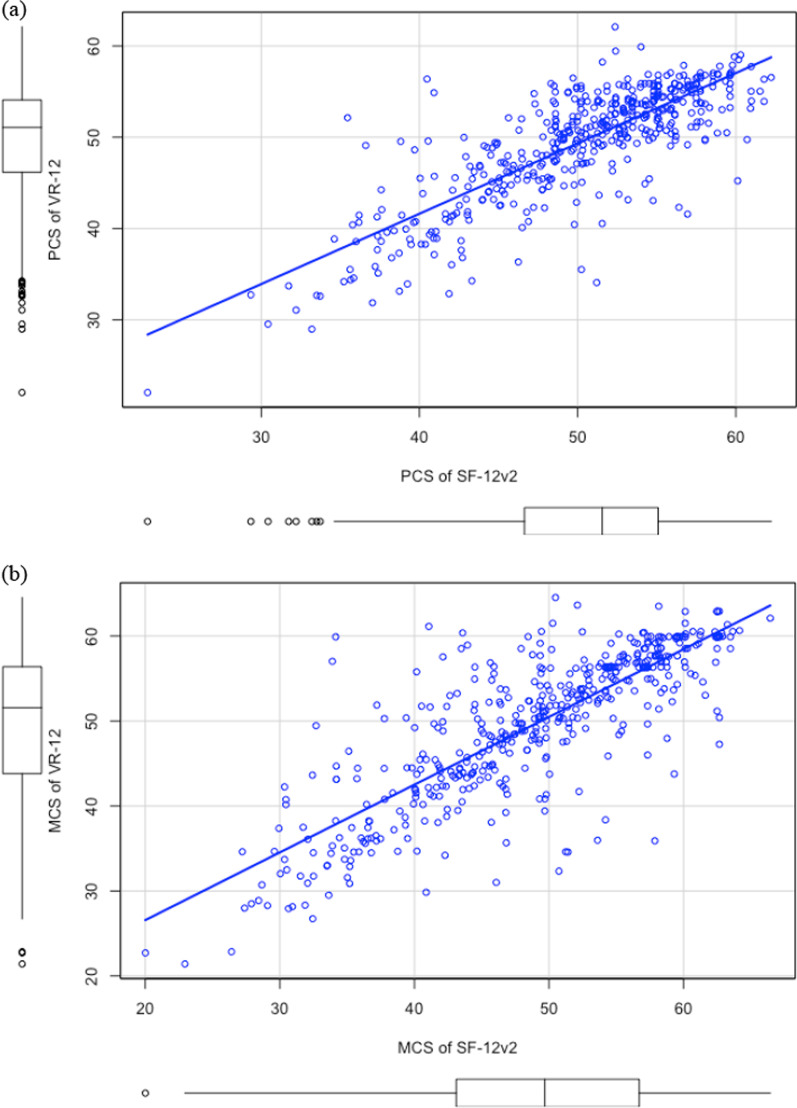
Scatter plots of **a** the physical component score (PCS), and **b** the mental component score (MCS), between the SF-12v2 and the VR-12

Table 3 provides the mean and SD for the component and scale scores of the two instruments and their differences. The mean PCS of the SF-12v2 and the VR-12 were 50.3 and 49.6, respectively. The mean MCS of the SF-12v2 and the VR-12 were 49.0 and 49.7, respectively. Among the eight scales, the GH scale showed the largest mean difference, whereas the VT scale showed the largest absolute difference (Table 3). The Spearman rank correlation between PCS of the two instruments was 0.78 and that between MCS of the two instruments was 0.80. Figure 1 presents the scatterplots of the two instruments for each component. For the eight scales, the polychoric correlation between the two instruments was the lowest for the SF scale (r = 0.68), followed by the RE scale (r = 0.73). The correlation for the other scales was at least 0.83 (Table 3).

**Table 3:** Descriptive Statistics of SF-12v2, VR-12 and the paired differences, SF-12v2 – VR-12

	SF-12v2		VR-12		SF-12v2—VR-12		|SF-12v2—VR-12|		Correlation between SF-12v2 and VR-12*
	Mean (SD)	Range	Mean (SD)	Range	Mean (SD)	Range	Mean (SD)	Median	
PF	91.2 (18.9)	0, 100	91.6 (18.5)	0, 100	− 0.45 (10.3)	− 50.0, 50.0	3.05 (9.9)	0	0.94
RP	80.9 (21.6)	0, 100	82.2 (21.7)	0, 100	− 1.3 (21.0)	− 100, 100	9.9 (18.6)	0	0.68
BP	80.0 (21.1)	0, 100	81.3 (20.2)	0, 100	− 1.4 (12.7)	− 75.0, 50.0	5.7 (11.5)	0	0.89
GH	58.0 (24.3)	0, 100	61.6 (20.9)	0, 100	− 3.6 (12.9)	− 60.0, 50.0	7.2 (11.3)	0	0.87
VT	61.9 (23.4)	0, 100	63.9 (25.4)	0, 100	− 2.0 (16.0)	− 100, 60.0	11.2 (11.6)	10.0	0.84
SF	86.0 (19.5)	0, 100	84.8 (20.2)	0, 100	1.1 (13.7)	− 75.0, 75.0	8.5 (16.6)	0	0.85
RE	79.4 (21.4)	0, 100	82.1 (21.1)	0, 100	− 2.7 (18.4)	− 100, 100	8.5 (16.6)	0	0.73
MH	70.8 (18.8)	12.5, 100	72.3 (17.3)	10.0, 100	− 1.5 (11.4)	− 42.5, 50.0	8.3 (8.0)	5.0	0.83
PCS	50.3 (6.5)	22.8, 62.2	49.6 (6.2)	22.0, 62.1	0.7 (4.0)	− 16.7, 17.1	2.9 (2.8)	1.9	0.78
MCS	49.0 (9.0)	20.0, 66.4	49.7 (8.8)	21.4, 64.5	− 0.7 (5.4)	− 25.8, 22.0	3.6 (4.1)	2.0	0.80

*SD* standard deviation, *PF* physical functioning, *RP* role physical, *BP* bodily pain, *GH* general health, *VT* vitality, *SF* social functioning, *RE* role emotional, *MH* mental health, *PCS* physical component score, *MCS* mental component score

*Polychoric correlation was reported for the eight scales, and Spearman rank correlation was reported for the PCS and the MCS

**Fig. 2:**
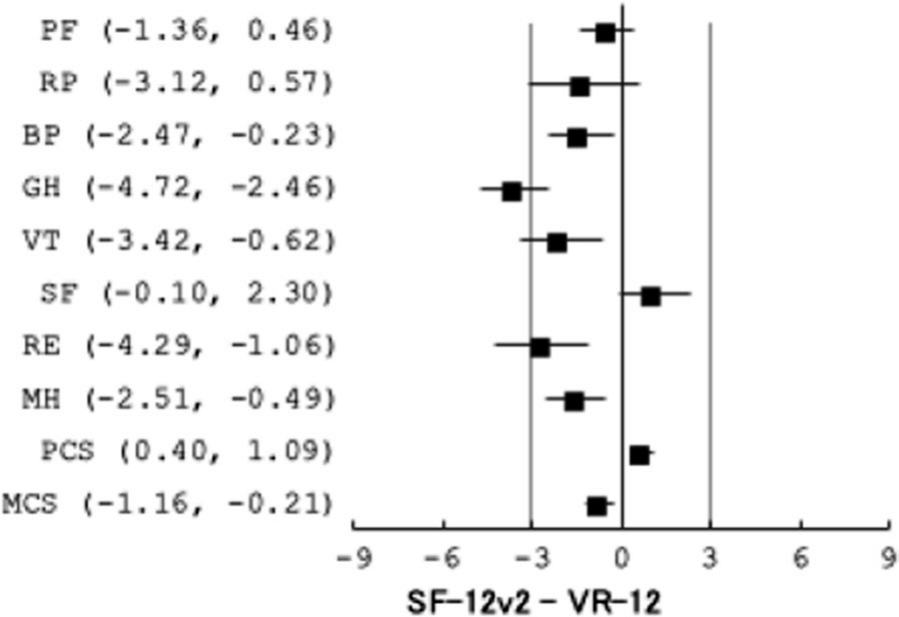
95% confidence intervals for the pair differences of the two component and eight scale scores of the SF-12v2 and VR-12

Figure 2 shows the 95% CIs for the paired differences of the component and scale scores of the two instruments. The average paired difference between the PCS of the SF-12v2 and that of the VR-12 (SF- 12v2—VR-12) was 0.7 for the PCS, 95% CI (0.4–1.1), which fell within its MCID of 3. For the MCS, the average difference was − 0.7, 95% CI (− 1.2 to − 0.2), which also fell within its MCID of 3. Among the eight scales, only the PF, BP and MH scales had their 95% CIs fell entirely within the MCID of 3 (Fig. 2).

**Fig. 3:**
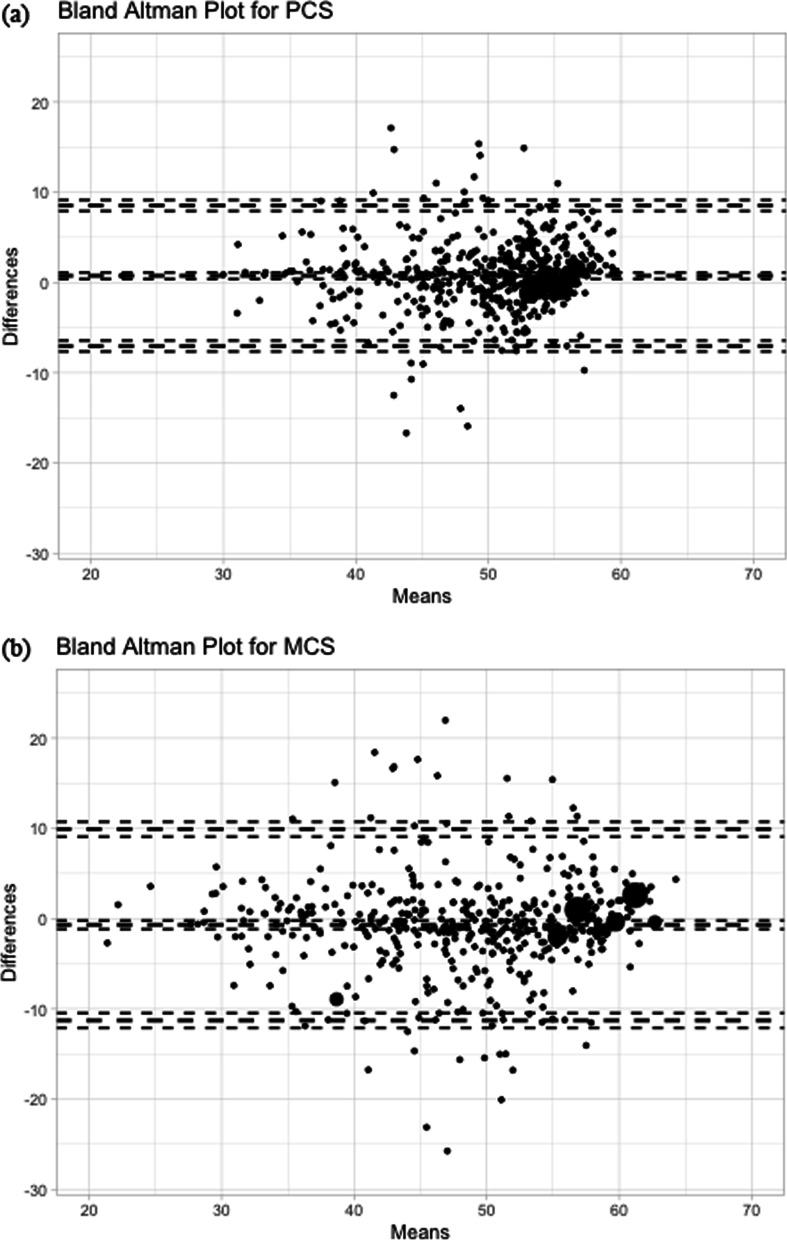
Bland–Altman Plots for the differences of **a** the physical component score (PCS), and **b** the mental component score (MCS), between the SF-12v2 and the VR-12

**Fig. 4:**
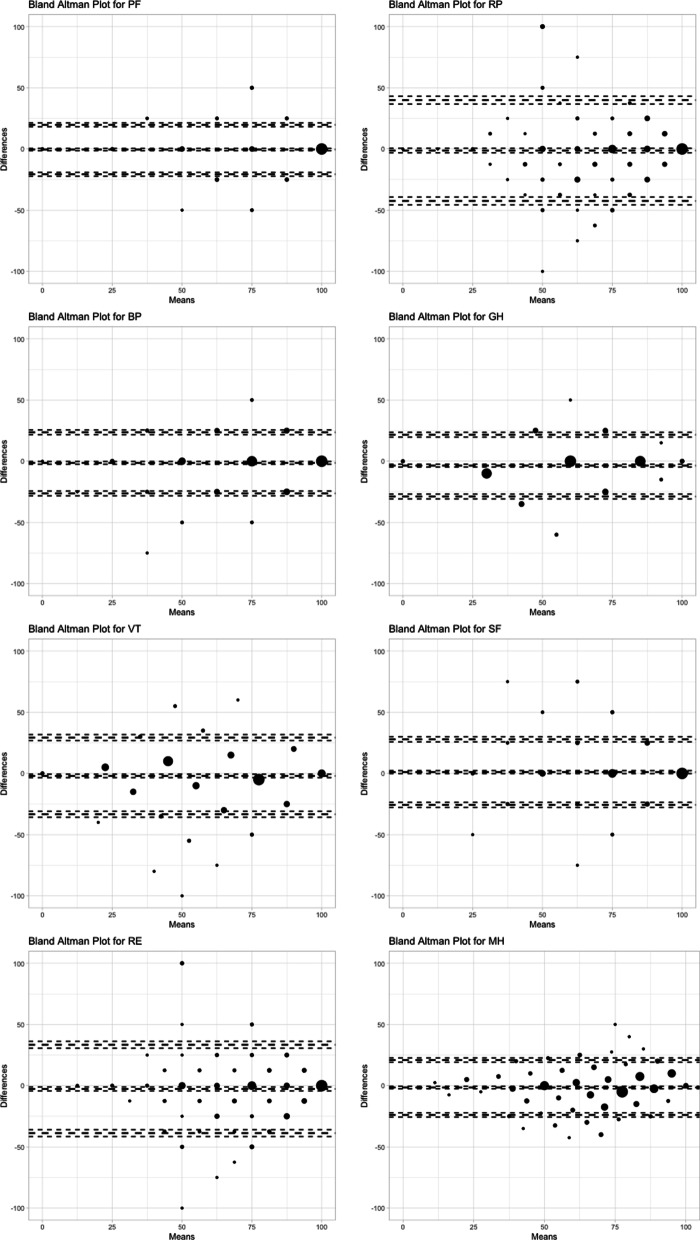
Bland–Altman Plots for the differences of the eight scales, between the SF-12v2 and the VR-12

The Bland–Altman plots for assessing the agreement between the PCS and the MCS of the two scales are shown in Fig. 3. The 95% limits of agreement for the PCS were − 7.0, 8.5, which fell outside the MCID of 6 for individual responses. For the MCS, the 95% limits of agreement were − 11.2, 9.9, which also fell outside the MCID of 7 for individual responses. Figure 4 depicts the Bland–Altman plots for all the eight scales. Their lower and upper limits of agreement were at least − 20.7 and 19.8, respectively.

Table 4 shows the potential factors of the differences between the PCS and the MCS of the SF-12v2 and the VR-12. Examining the residuals did not reveal a substantial model inadequacy. Those with a higher age (estimate = − 0.03; *p* = 0.027) or separated/divorced/widowed (estimate = − 1.6; *p* = 0.029) reported a significantly lower PCS from the SF-12v2 than from the VR-12. However, the effect sizes were all within the two MCID units for the PCS. No significant factors were found for the MCS.

**Table 4:** Univariable analysis for factors associated with the paired differences, SF-12v2 – VR-12

Variables	Physical component score (PCS)			Mental component score (MCS)		
	Estimates	95% CI	*P* value	Estimates	95% CI	*P* value
Age (years)	− 0.03	(− 0.06, − 0.00)	0.027*	− 0.0002	(− 0.04, 0.04)	0.993
Sex						
Male (Ref)	0	/	/	0	/	/
Female	− 0.08	(− 0.82, 0.66)	0.840	0.52	(− 0.49, 1.52)	0.313
Marital status			0.041			0.453
Single (Ref)	0	/	/	0	/	/
Married/Cohabiting	− 0.73	(− 1.48, 0.01)	0.055	0.65	(− 0.37, 1.68)	0.209
Separated/Divorced/Widowed	− 1.61	(− 3.04, − 0.17)	0.029*	0.40	(− 1.57, 2.37)	0.690
Educational level						
Primary school or below (Ref)	0	/	/	0	/	/
Secondary	− 0.75	(− 2.41, 0.91)	0.377	− 1.16	(− 3.43, 1.11)	0.315
Bachelor or above	0.20	(− 1.47, 1.87)	0.811	− 0.74	(− 3.03, 1.54)	0.523
Occupation						
Employed/In the working force (Ref)	0	/	/	0	/	/
Not in working force	− 0.41	(− 1.16, 0.32)	0.267	− 0.23	(− 1.24, 0.78)	0.653
Chronic Illness						
No (Ref)	0	/	/	0	/	/
Yes	0.62	(− 0.29, 1.53)	0.178	0.18	(− 1.06, 1.42)	0.773

*P value < 0.05

## Discussion

To the best of our knowledge, this is the first study to investigate the differences between the SF-12v2 and the VR-12 in the Chinese population, and the first to use the Bland–Altman analysis to measure the extent of agreement between the SF-12v2 and the VR-12 on an individual basis. We found no discernible average differences between the PCS, MCS and some scales of the SF-12v2 and the VR-12. However, there were substantial individual differences.

Despite differences in item format and type between the SF-12v2 and the VR-12, this study found no average differences between the PCS and the MCS of the SF-12v2 and the VR-12, based on the MCID of 3 for both component scores. This supports the results of a previous study that the two component scores of the MOS SF-12 and the VR-12 yielded similar results when comparing patients with osteoarthritis and those with focal cartilage defects, although the MOS SF-12 version 1 was used instead of the version 2 used here [35]. Moreover, a group of patients who underwent spinal treatment completed the MOS SF-36 and had its PCS and MCS compared with the corresponding VR-12 component scores that were converted from the MOS SF-36 using a conversion algorithm from the Boston University School of Public Health. The PCS and the MCS of the MOS SF-36 were strongly correlated with the corresponding converted VR-12 component scores (correlation coefficient = 0.85–0.97) [36]. The correlation was higher than that in our sample when the SF-12v2 was used, which was 0.78–0.80. The lower observed association in our sample may be due to the use of much fewer items resulting in a greater variability. Nevertheless, the SF-12v2 and VR-12 remains strongly associated in their two component scores. The equivalence of the average PCS and MCS between the two instruments facilitates the use of either instrument to compare group differences. Moreover, including studies using either instrument in a meta-analysis or systematic review would not induce substantial study heterogeneity.

Among the eight scales, no average differences were also found in the PF, BP, SF and MH scales. However, the other four scales, RP, GH, VT and RE, did not have their 95% confidence intervals fall entirely within the 3 units of average equivalence limits. For both the RP and RE scales, item responses from the VR-12 require reverse code but those from the SF-12v2 do not. For GH, the item responses from the SF-12v2 and the VR-12 are coded differently. Moreover, the VT scale comprises only one item which was scored on a 5-point Likert scale in the SF-12v2 but on a 6-point Likert scale in the VR-12. Such method effects may have contributed to the systematic differences in their scale scores between the SF-12v2 and the VR-12. However, the MH scale had both of its items share the same difference in scoring as in the item of the VT scale. The observed average equivalence of the MH scale but not the VT scale may be because the MH scale comprises more items than the VT scale. All the PF, BP and SF scales between the SF-12v2 and the VR-12 share the same scoring methods, with generally only minor differences in item wording. Item 2a is the only item having a noticeable cultural difference in terms of the activities considered, with practicing Tai-Chi in the SH-12v2 and playing golf in the VR-12. Interestingly, the PF scale that includes item 2a did not show an average difference. This may be due to both golf and Tai-Chi being common activities nowadays.

Based on the Bland–Altman analysis, there were large individual differences in both the PCS and the MCS of the SF-12v2 and the VR-12 when compared with the MCIDs for individual responses of the two components. We explored the potential factors contributing to these differences, but none of them showed a substantial impact on the response differentials. In general, self-reported responses, as opposed to objective measurements, carry higher variability because of the extra intrapersonal variation even when the underlying construct remains stable. Therefore, a self-reported instrument would often be considered unreliable when comparing individual responses from specific individuals [37]. This echoes a much larger MCID when comparing individual responses. For example, the MCID for PCS is 3 for group comparisons but 6 for individual comparisons, while that for MCS is 3 for group comparisons and 7 for individual comparisons [31]. In our context of comparing the SF-12v2 and the VR-12, the difference in item format and type as well as scoring mechanism could have added variation to the individual responses, possibly resulting in a larger difference in the component scores between the two instruments. Hence, comparing the individual scores of the two components of the SF-12v2 and the VR-12 is not recommended.

All the eight scales showed individual differences between the SF-12v2 and the VR-12. Despite the eight scales do not possess a MCID for individual responses, their limits of agreement were much wider than those of the PCS and the MCS. Among the eight scales, the PF scale had the shortest limits of (− 20.7, 19.8), which are much beyond the MCIDs for individual responses of 6 and 7, respectively, for the PCS and the MCS. Indeed, it is known that not all scales have performed well in Chinese [26, 27, 32]. The MH scale of the SF-12v2 was shown to have low internal consistency [26]. Moreover, several scales did not possess equivalence between the English and Chinese languages, nor resemble the corresponding scales in the SF-36v2 [26, 27]. In general, there was also more interest on the two component scores [6, 38], and there should be cautious use of the eight scales with a good understanding of their limitations.

There are several limitations to this study. First, we have only assessed the equivalence in the estimates obtained from the two instruments. Assessing the structural equivalence may provide stronger evidence in terms of comparable validity and interpretation. However, items of the two instruments may not have the same number of response choices nor the same scoring scheme. Such differences may pose challenges to the application of multiple group confirmatory factor analysis and differential item functioning for assessing the structural equivalence. Second, it will be useful to compare the two instruments with external criteria for determining which one is better. For instance, the comprehensive item bank of physical function items from the Patient-Reported Outcomes Information System can be a useful criterion that future studies may consider [39]. Third, we have not considered newer 2009 norms and 2000–2002 norms for scoring the SF-12v2 and the VR-12, respectively. Future studies that examine the comparisons using the new norms would be useful. Fourth, we did not randomize the order of administering the SF-12v2 and the VR-12. The participants had to complete 52 other items before completing the VR-12 that may reduce the chance of recalling the earlier responses, the order effect may induce systematic differences and thus confound the observed differences between the two instruments. Further studies that randomize the order of administration would be desirable. Fifth, we focused on the Chinese versions of the SF-12v2 and the VR-12. Similar comparisons on other language versions, would be desirable to assess the generalizability of our results.

## Conclusion

The two component scores PCS and MCS of the SF-12v2 and the VR-12 are equivalent when they are used in group comparisons. While the SF-12v2 has been well developed and tested in many ethnic groups, the VR-12 is a low-cost alternative. However, they have considerable individual differences, and caution should be exercised when comparing individual responses for their use in clinical practice.

## Data Availability

The datasets generated and/or analyzed in this study are not publicly available due to confidentiality.
